# Single pyrimidine discrimination during voltage-driven translocation of osmylated oligodeoxynucleotides via the α-hemolysin nanopore

**DOI:** 10.3762/bjnano.7.11

**Published:** 2016-01-22

**Authors:** Yun Ding, Anastassia Kanavarioti

**Affiliations:** 1 Chemistry Department, University of Utah, Salt Lake City, UT, USA; 2 Yenos Analytical LLC, El Dorado Hills, CA, USA

**Keywords:** DNA sequencing, α-hemolysin, ion-channel measurements, nanopore, osmium tetroxide bipyridine, osmylated oligos, osmylation, single-stranded DNA (ssDNA)

## Abstract

The influence of an electric field on an isolated channel or nanopore separating two compartments filled with electrolytes produces a constant ion flux through the pore. Nucleic acids added to one compartment traverse the pore, and modulate the current in a sequence-dependent manner. While translocation is faster than detection, the α-hemolysin nanopore (α-HL) successfully senses base modifications in ssDNA immobilized within the pore. With the assistance of a processing enzyme to slow down translocation, nanopore-based DNA sequencing is now a commercially available platform. However, accurate base calling is challenging because α-HL senses a sequence, and not a single nucleotide. Osmylated DNA was recently proposed as a surrogate for nanopore-based sequencing. Osmylation is the addition of osmium tetroxide 2,2’-bipyridine (OsBp) to the C5–C6 pyrimidine double bond. The process is simple, selective for deoxythymidine (dT) over deoxycytidine (dC), unreactive towards the purines, practically 100% effective, and strikingly independent of length, sequence, and composition. Translocation of an oligodeoxynucleotide (oligo) dA_10_XdA_9_ via α-HL is relatively slow, and exhibits distinct duration as well as distinct residual current when X = dA, dT(OsBp), or dC(OsBp). The data indicate that the α-HL constriction zone/β-barrel interacts strongly with both OsBp and the base. A 23 nucleotide long oligo with four dT(OsBp) traverses 18-times slower, and the same oligo with nine (dT+dC)(OsBp) moieties traverses 84-times slower compared to dA_20_, suggesting an average rate of 40 or 180 μs/base, respectively. These translocation speeds are well above detection limits, may be further optimized, and clear the way for nanopore-based sequencing using osmylated DNA.

## Introduction

Nanopore technology has seen great advancements following a rather slow start as a global analytical tool for anything from cells to single molecules [1–2]. Progress in X-ray crystallography to deduce structural details of protein pores [3–5], and progress in manufacturing of nanometer-sized holes in thin layers of inorganic materials [6–9] has led to exploration of both natural and synthetic pores for a number of applications [10–11]. The concept, reintroduced independently by David Deamer and George Church in the context of DNA sequencing, is based on applying a potential across an open pore embedded in an insulating surface that separates two compartments filled with electrolyte. A nucleic acid in one compartment can move through the pore to the other compartment influenced by the electric field and the interactions with the pore, and concurrently modulate the current. Protein pores such as the α-HL, a modified version of the *Mycobacterium smegmatis* porin A (MspA), and the Ph29 connector channel have been investigated as single molecule sensing devices for ssDNA, RNA, dsDNA, and proteins [12–14]. The advantage of the natural pores is that they are well defined and highly reproducible, whereas the advantage of the solid-state nanopores is that they can be manufactured to desirable dimensions. Sensing rare DNA bases produced by UV-radiation and other oxidative processes was successfully accomplished by chemically modifying the base and/or immobilizing the strand within the α-HL pore [15–17]. Such measurements revealed that current obstruction upon passing of a telomere DNA via the pore yields folding information [18]. The observation that α-HL can distinguish between RNA and DNA homopolymers [19–21] led to a large effort in government, academia, and industry to explore nanopores for DNA sequencing [22–27].

Early on it became evident that translocation of ssDNA, at about 1 to 2 μs per base, is too fast to report back on the small current modulation associated with the passing of each base [13]. It also became clear that the protein pores, such as α-HL and MspA, have sensing regions that interact with a sequence and not a single base [13,28–30]. Attempts to overcome these challenges include modification of the wild type proteins by site-directed mutagenesis or other means to improve/narrow the sensing region [30–32], tagging of the bases by amino acids or by PEGylation [33–35], assessing the specific current level sensed by the pore for each possible sequence in order to create a complete data set of current signatures [36], and the use of improved bioinformatic tools [37–38]. Incorporation of a processing enzyme, phi29 DNA polymerase, at the top of the pore to move the nucleic acid one base at a time, was a game changer because it slowed down translocation to the ms level [39–41]. Translocation speed in the presence of an enzyme is reduced by three to four orders of magnitude and it is two to three orders slower than necessary for detection, yielding a tentative 100 to 1000-fold reduction in reading speed compared to an optimal situation [12–13]. In a nutshell progress in nanopore-based sequencing of DNA is still limited by three issues: (i) the chemical comparability of the four nucleobases in the context of current modulation, (ii) the fact that translocation in the absence of a processing enzyme is too fast for detection and in the presence of such enzyme too slow for reading genomes, and (iii) that so far no system detects and reports back a distinct signal that corresponds to a single base.

**Scheme 1:**
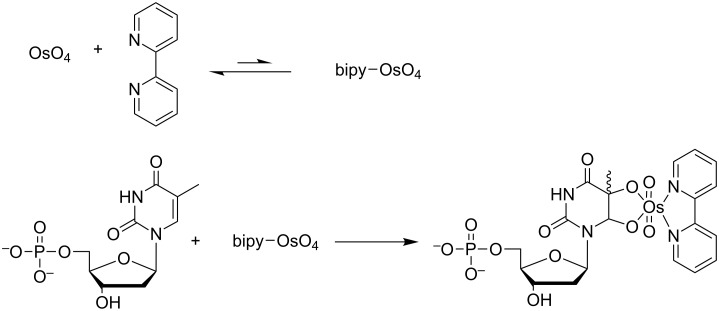
OsBp with dTMP. Reaction of osmium tetroxide with 2,2’-bipyridine forms a reactive complex (OsBp or bipy–OsO_4_), which in a second step reacts with a pyrimidine (thymidine monophosphate (dTMP) shown here) to form the osmylated pyrimidine [42,44]. One way to illustrate the difference between osmylated and intact bases is to compare (molecular weight) of each: dC (111), dT (126), dA (135), dG (151); dC-OsBp (521), dT-OsBp (536), i.e., osmylation adds about 400% mass to the reactive base compared to the unreactive one.

**Figure 1:**
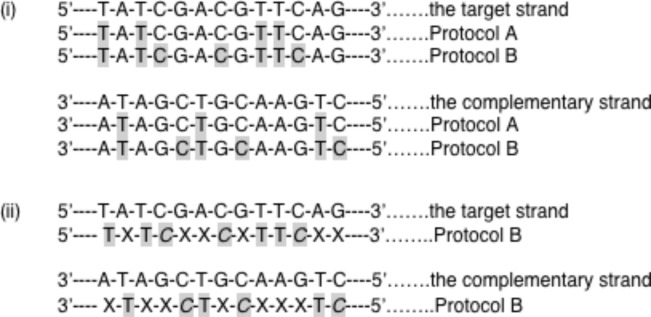
Strategy for sequencing DNA using the osmylated strands. All sequences shown refer to deoxybases; for simplicity d is left out. (i) Original proposed route for obtaining the sequence of the target strand by osmylating both the target strand and its complementary with Protocols A and B [46]; Protocol A yields primarily osmylated-T (shaded) and Protocol B yields practically 100% osmylated pyrimidines (shaded T and C). Sequencing the complementary strand provides indirectly information on A and G. This approach presumes discrimination between osmylated from intact base, but no discrimination among pyrimidines. (ii) The data presented in this report strongly suggest that α-HL discriminates osmylated-T (shaded regular font) from osmylated-C (shaded italic font) and intact purine (X). Therefore a streamlined strategy might be to sequence osmylated strands using only one protocol, perhaps Protocol B. For further discussion on the sequencing strategy [47].

Minimal effort has been placed so far in labeling one or more of the bases with a moiety to add bulkiness and impose discrimination, perhaps, because selective labels are hard to find and because proof of efficient labeling of a gigabase long nucleic acid is seen as an unattainable goal. While working with metalorganic molecules to label ssDNA, we evaluated OsBp [42]. OsBp is known to add to the C5–C6 double bond of the pyrimidine ring (Scheme 1). Because osmium is a good contrast agent for imaging by electron microscopy (EM), osmylated DNA was proposed 60 years ago and exploited in attempts to obtain DNA sequence information by EM imaging [43–45]. The more recent advancement of nanopores as single molecule detection devices and the corresponding progress in manufacturing, parallelization, and commercialization of such platforms [24], supported the idea of testing osmylated DNA as a surrogate for nanopore-based sequencing (see Figure 1 and [46–47]).

**Figure 2:**
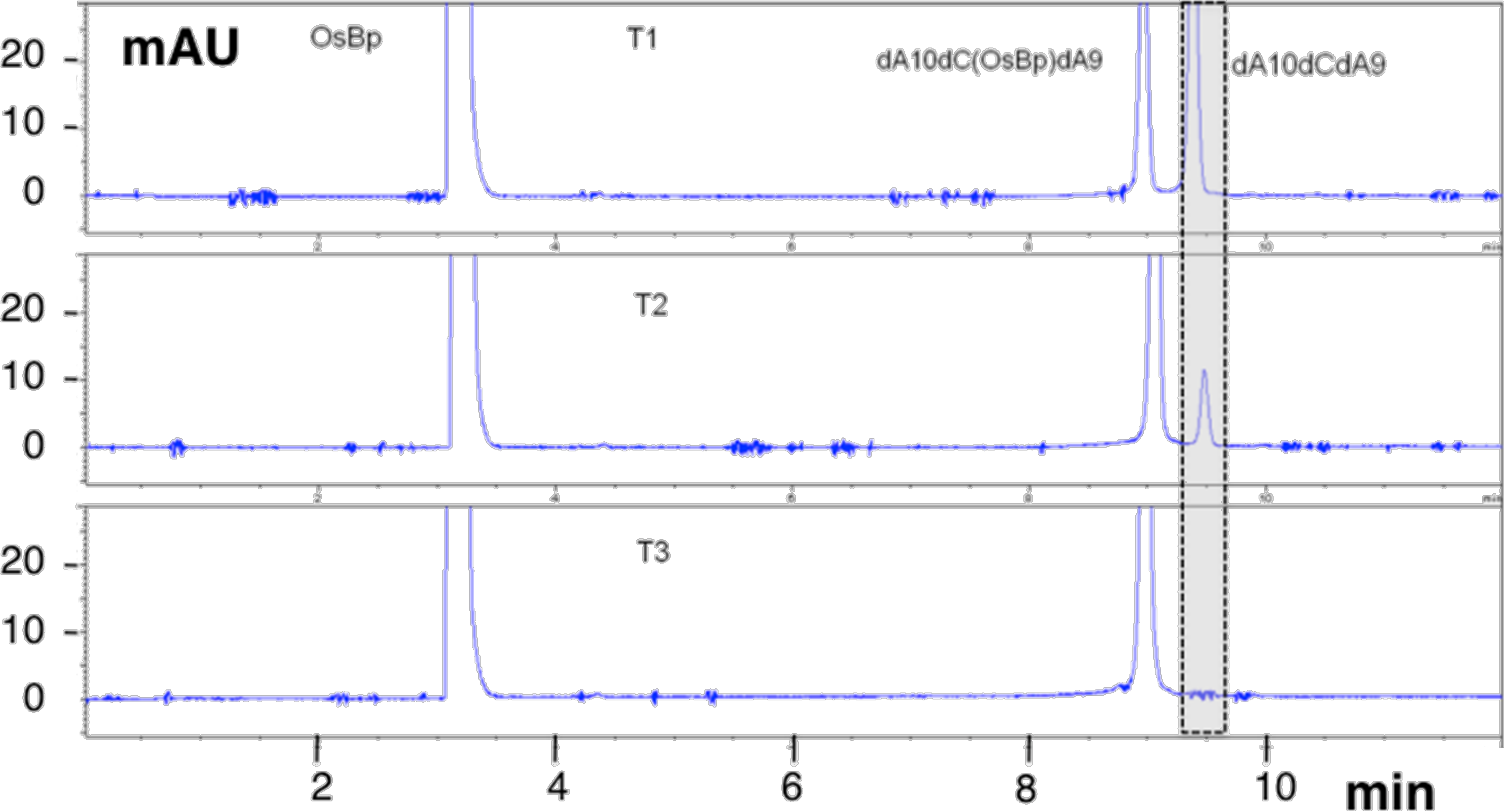
Reaction of 12.6 mM OsBp (the highest concentration of OsBp used under our conditions) with 20 μM dA_10_dCdA_9_ monitored automatically by CE (see Experimental section, Part A). Reactants were mixed in a CE vial and aliquoted/analyzed automatically by the instrument. T1 represents 6 min, T2 24 min and T3 76 min incubation after mixing. Signal is shown at 260 nm. Reaction mixture was incubated at 25 ± 1 °C within the thermostated autosampler of the instrument. Shaded peak is the original oligo that migrates last, OsBp is neutral and migrates early. The reaction product (osmylated oligo) migrates ahead of the intact oligo. Oligo dA_10_dCdA_9_ used in this reaction was PAGE-purified with confirmed purity by CE at 97%. The small spikes, at about 1 mAU with no steady migration time, are due to noise; the peak height of both oligo and osmylated oligo reach 120 mAU.

The proposition was motivated by studies showing that the osmylation [48] is a remarkably clean reaction yielding the expected products in practically 100% yield with no detectable side-reactions. An example is provided in Figure 2 where the reaction between OsBp and dA_10_dCdA_9_ is monitored by capillary electrophoresis (CE) analysis. Any side-reaction, or any backbone degradation, or oxidative damage would have been detectable as additional peaks with typical detectability of 0.1%. Any product formed by these processes is expected to retain some nucleotides, and therefore be spectrophotometrically visible. Indeed the only detectable reaction is the conversion of dA_10_dCdA_9_ to dA_10_dC(OsBp)dA_9_. Evidence for the addition of one unit OsBp per pyrimidine double bond has been reviewed in the literature [43–45] and was specifically tested under our conditions by UV–vis, and ^1^H NMR [42]. Moreover molecular masses corresponding to adducts with 1, 2, or 3 OsBp moieties were obtained by MALDI TOF for oligos with 1, 2, or 3 Ts, respectively [42].

Unpublished data suggest false positives and false negatives to be below 1/10,000, a remarkable feat for any modification reaction. The reactivity is not impeded by long sequences of pyrimidines, as evidenced by the rate for complete osmylation of dT_15_ that is, within experimental error, comparable to the rate of monomer osmylation, i.e., dTTP to dTTP(OsBp) [46]. It turns out that the same protocol, in the absence of any denaturing agents, works predictably and reproducibly for short and long oligos, as well as for M13mp18, a circular 7459 bases long ssDNA with secondary structure [46]. The success in using the same protocol for ssDNA with secondary structure as for short oligos is attributed to the hydrophobicity of the OsBp moiety that disrupts base stacking interactions [49]. This feature implies that any ssDNA of unknown sequence can be predictably osmylated. Additionally osmylated oligos and osmylated ssDNA are stable at room temperature for days as shown by CE analysis both in the presence of OsBp, or after purification/removal of the excess label [42,46].

**Figure 3:**
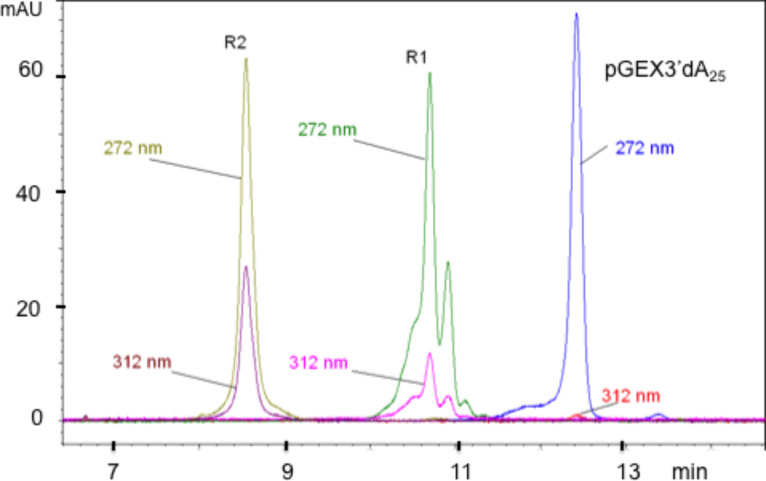
Capillary electrophoresis (CE) overlapping traces of oligodeoxynucleotides pGEX3’-dA_25_ intact and pGEX3’-dA_25_ at R1 and R2 levels of osmylation per protocols A and B, respectively (Experimental section, Part A). Materials are at comparable, but not identical, concentrations. Migration is in the order of intact oligo last, R2 early, and R1 in the middle. Traces are shown at two wavelengths, at 272 and 312 nm, to illustrate that DNA exhibits about 1% absorbance, whereas R1 and R2 absorb substantially, and R2 > R1. The detail in the R1 peak is attributed to different topoisomers produced from either top or bottom addition to the C5–C6 double bond. Topoisomers exist also with R2, but are too many to be resolved. The ratio R(312 nm/272 nm) represents a normalization, and the wavelengths are selected to maximize the value of R [46].

It was also shown that the osmylation product exhibits a new chromophore in the range 300 to 320 nm [42,46] where DNA does not absorb (see below in Figure 3). This chromophore was the basis for developing a UV–vis assay to quantitatively measure the extent of osmylation, and facilitate the development of the two protocols (Figure 1(i)); Protocol A exploits low concentration of OsBp with short incubation, and yields primarily dT(OsBp), and Protocol B uses higher concentration (12.6 mM) with longer incubation and yields practically 100% (dT+dC)(OsBp); both protocols work at room temperature. The UV–vis assay serves as a quality control assay to confirm extent of osmylation.

The realization that osmylation adds a 4-fold mass to the reacting base (Scheme 1, caption) fueled the speculation that any size-suitable nanopore could discriminate between osmylated and native base, and led to a proposed sequencing strategy (Figure 1). Preliminary experiments to assess pore size suitability using solid-state silicon nitride nanopores [50] showed that 1.6 nm wide pores permit translocation of 80-mer long osmylated oligos, and exhibit dramatic translocation slowdown with enhanced osmylation. These observations led us to undertake the α-HL nanopore measurements reported here.

## Results and Discussion

### Interaction of α-HL nanopore with single osmylated pyrimidine during oligo translocation

This is the first study to assess translocation properties of osmylated oligos via the α-HL nanopore. Based on chemical structure the expectation was that the constriction site of α-HL at 1.4 nm might be too narrow to allow translocation of osmylated oligos. Nevertheless experiments with solid-state nanopores at 1.6 nm diameter provided evidence for translocation [50], and so do the experiments with α-HL described below. Here we explored the translocation properties of a series of 20-mer oligoadenylates where the 11th nucleotide was a deoxypyrimidine, such as dT, dC, 5-Me-dC, and dU, as well as a 23-mer oligo consisting of all four bases. To explore the effect on oligo entry in the α-HL pore we also tested the later with a dA_25_ tail added either to the 3’- or the 5’-end (Table 1).

**Table 1:** List of oligos used in this study, their purity (see Experimental), total number of nucleotides, *N*_total_, and sequence. The data obtained can be found in Table 2.

ODN	% purity by CE	*N*_total_	sequence : deoxyoligonucleotide (5’—3’)

dA_20_	83	20	AAA AAA AAA AAA AAA AAA AA
dA_10_dTdA_9_	86	20	AAA AAA AAA ATA AAA AAA AA
dA_10_dCdA_9_	86	20	AAA AAA AAA ACA AAA AAA AA
dA_10_5-MedCdA_9_	74	20	AAA AAA AAA A5-MeCA AAA AAA AA
dA_10_dUdA_9_	78	20	AAA AAA AAA AdUA AAA AAA AA
pGEX3'	89	23	CCG GGA GCT GCA TGT GTC AGA GG
dA_25_-pGEX3’	88	48	(A)_25_ CCG GGA GCT GCA TGT GTC AGA GG
pGEX3’-dA_25_	95	48	CCG GGA GCT GCA TGT GTC AGA GG (A)_25_

The oligos used in this study were purchased from Integrated DNA Technologies, as PAGE-purified and desalted materials (Table 1). They were analyzed by capillary electrophoresis (CE) to assess purity, and then osmylated (see Experimental section); R1 refers to Protocol A and yields primarily dT(OsBp) and R2 refers to Protocol B and yields (dT+dC)(OsBp) oligos. The products were purified from excess OsBp in a spin-minicolumn, and analyzed by CE to confirm the extent of osmylation (see below Table 2, 2nd column, and Figure 3), as well as the absence of unreacted OsBp. The modified oligos were evaluated via the α-HL pore using an instrument custom made for the Chemistry Department of the University of Utah equipped with a glass pore membrane (GNM, see Experimental section). Ion channel measurements were done using conditions for unmodified oligos as described in [14]: 10 μM oligo in 1.00 M KCl, at pH 7.4 with 10 mM potassium phosphate buffer at 22 ± 1 °C. Up to four different probing voltages, namely 100, 120, 140, and 160 mV (*trans* vs *cis*) were tested.

**Figure 4:**
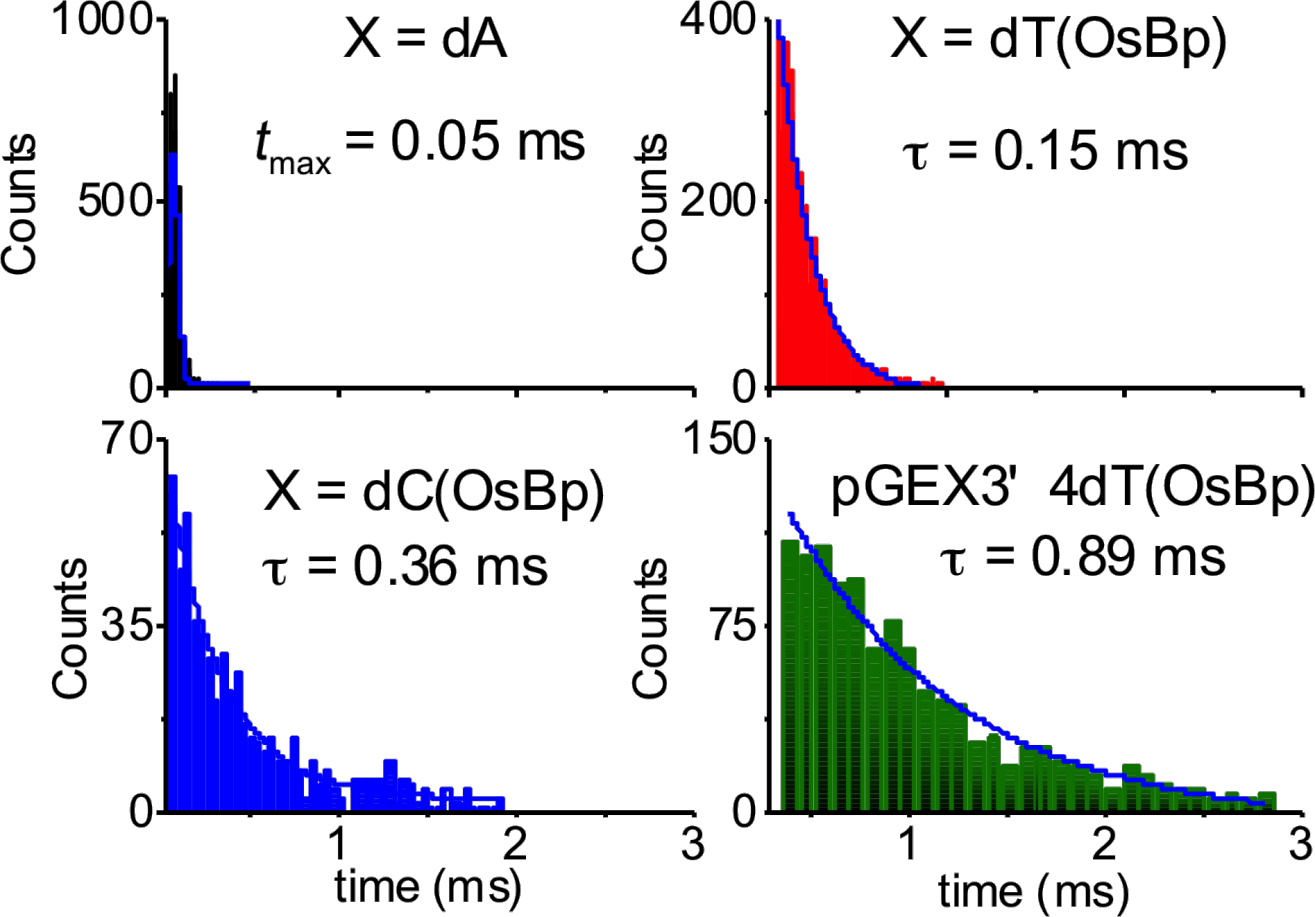
Translocation time histograms for four different oligos: Three are oligodeoxyadenylates, dA_10_XdA_9_ where X = dA, dT(OsBp), or dC(OsBp). The fourth is a 23 nt long deoxyoligo pGEX3’ (PCR primer) and contains four modified bases, dT(OsBp). Translocation speed decreases in the presence of a single modified pyrimidine; dC slows down the oligo more compared to dT, and the presence of additional osmylated bases yields further slow-down.

The observation of decreasing dwell times with increasing voltage (Table 2) is strong evidence that the osmylated oligo translocated. Five 20 nt long oligodeoxyadenylates were screened: dA_20_ serving as control, and four dA_10_XdA_9_ where X is an osmylated pyrimidine: X = dT(OsBp), dC(OsBp), 5-MedC(OsBp), or dU(OsBp). Translocation time for dA_20_ was obtained from the maximum value of a Gaussian curve analysis of the data and values at different voltages were as expected for this set up (Table 2, first entry). Translocation data from the other oligos were fitted with a first-order exponential decay, and provided translocation times, τ, also illustrated in Figure 4 and listed in Table 2. Typically current levels (*I*_0_) in unobstructed pores are about 120 ± 5 pA at 120 mV (conductance ≈ 1000 pS) at our conditions. Normalized values of residual current (*I*_r_/*I*_0_) were obtained from the maximum value of the current histograms and are also reported in Table 2; they are accurate to ±1%, and did not vary with voltage.

**Table 2:** List of Oligos used in this study together with the number of Ts and Cs osmylated bases. R1 or R2 (at 312 nm/272 nm) is given by the ratio of the peak area at the two different wavelengths following protocol A or protocol B, respectively. Sequence and purity of each oligo can be found in Table 1. Normalized current obstruction, *I*_r_/*I*_0_, (*trans* vs *cis*). Translocation time, *t* or τ, is obtained from the events-vs-time histogram. Experimental conditions: 10 μM DNA in 1.0 M KCl, 10 mM PBS, pH 7.4, 22 ± 1 °C.

material	R1 or R2	number of osmylated bases	*I*_r_/*I*_0_ (±1%) at 120 mV	translocation time, τ (ms)			
				100 mV	120 mV	140 mV	160 mV

dA_20_	0.01	0	0.14	0.07	0.05	0.03	a
dA_10_dTdA_9_	0.11	1 (T)	0.08	0.20	0.15	0.10	—
dA_10_dCdA_9_	0.11	1 (C)	0.11	—	0.36	0.26	0.24
dA_10_5-MedCdA_9_	0.11	1(5-MeC)	0.12	—	0.31	0.24	0.20
dA_10_dUdA_9_	0.11	1 (dU)	0.12	—	0.47	0.36	0.30
pGEX3', R1	0.42	4 (T)	0.06	—^b^	0.89	0.49	0.35
pGEX3', R2	0.77	9 (T+C)	0.03	6.45	4.20	3.50	—
dA_25_-pGEX3’, R1	0.20	4 (T)	0.10	—^b^	0.65	0.45	0.36
pGEX3’-dA_25_, R1	0.20	4 (T)	0.11	—^b^	0.37	0.28	0.17
pGEX3’-dA_25_, R2	0.43	9 (T+C)	0.07	—^b^	0.53^c^	0.39^c^	0.17^c^

^a^Too fast to be analyzed with a 100 kHz filter and 500 kHz acquisition rate; ^b^event frequency is too low to allow population analysis in a reasonable amount of time; ^c^there are three different populations, the number here shows the dwell time for the first population (<10 ms).

**Figure 5:**
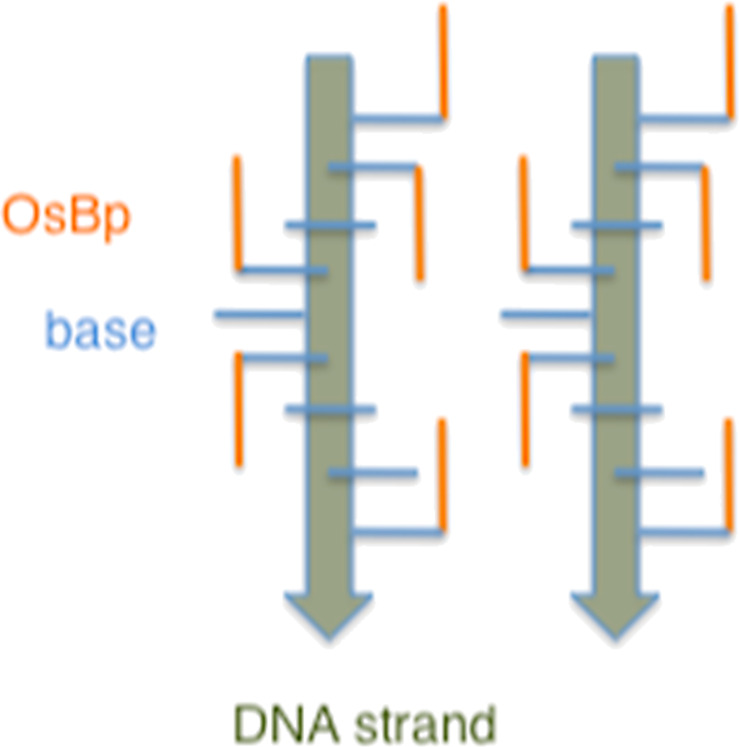
Representation of osmylated DNA strands to illustrate the practically parallel line-up of the OsBp moieties to the direction of the strand. This picture also demonstrates the plausible complexation of two strands by hydrophobic interactions between the OsBp moieties. Note that OsBp can add from the top or the bottom of the C5–C6 double bond, and vary from molecule to molecule (not shown here). Complexation is favored in solutions at relatively high oligo and high salt concentration.

Evidence for translocation of osmylated oligos via the α-HL pore, despite the apparent bulkiness of the OsBp moiety, was initially surprising. Actually the approximately orthogonal positioning of the nucleobases to the strand axis, and the top or bottom addition of OsBp to the pyrimidine double bond, lead to the conjecture that OsBp stretches parallel to the strand direction (Figure 5) and not perpendicular to it, which would enlarge the perimeter of this modified DNA. As speculated [46] the presence of the OsBp moiety reduced the residual current and slowed down the translocation of the modified oligo. However the markedly different ion-channel measurements observed between dA_10_dT(OsBp)dA_9_ and dA_10_dC(OsBp)dA_9_ were unexpected (see Graphical Abstract, Figure 4, and Table 2). It turns out that dT(OsBp) yields more relative current obstruction, but faster translocation compared to dC(OsBp). The observation that electrophoretic properties associated with a single modification in an oligo are so different is unprecedented, and strongly suggests interaction between the pore, most likely the constriction site of α-HL, with both the OsBp moiety and the nucleobase. It is not clear whether or not those interactions are direct or indirect via the corresponding solvation shells. It is plausible that the presence of OsBp inside the constriction site provides a highly confined environment in which the modified nucleotide is forced to rearrange to a less favored configuration as well as to a different solvation shell that “carries along” only what is critically important; all these changes are then detected as current modulation and slower dwell time.

In an attempt to understand the source of the differences between dT(OsBp) and dC(OsBp) we included dA_10_5-MedC(OsBp)dA_9_ and dA_10_dU(OsBp)dA_9_, expecting that 5-MedC would perhaps mimic dT and dU would mimic dC. Even though current modulation is comparable among 5-MedC, dC, and dU, dwell times are substantially different and in the order dU > dC > 5-MedC > dT >> dA with τ = 0.47, 0.36, 0.31, 0.15 and 0.05 ms at 120 mV (*trans* vs *cis*), respectively. These differences are well above experimental error, they are observed at all three different voltages, i.e., at 120, 140 and 160 mV (see Table 2), and clearly suggest a forceful interaction between the base and the nanopore for all four osmylated bases. As seen in Table 2 the residual current is 2 to 3% more blocking for osmylated dC, 5-MedC, and dU, and 6% more blocking for osmylated dT compared to intact dA. These differences appear small when compared to differences observed for immobilized bases within the pore [16]. It is noteworthy that in the present experiments the measured current modulation is “averaged” over the full sequence of the oligo. Hence it is reasonable to expect that discrimination at the single base level of an immobilized osmylated oligo would be larger than what is observed here, and it might not lead to overlap as seen in the current histogram on the TOC graphic.

### Dramatic translocation slow-down of osmylated oligos

At 120 mV the translocation speed of the oligoadenylate is reduced by a factor of 3, when the middle nucleotide (nt) is replaced by dT-OsBp (duration increased from 0.05 to 0.15 ms), and by a factor of 7, when it is replaced by dC(OsBp) (duration increased from 0.05 to 0.36 ms). To explore how osmylation affects translocation of a typical oligo, we tested pGEX3’, a 23 nt PCR primer, as well as two 48 nt long oligos with the sequence of pGEX3’ and an added dA_25_ tail either at the 3’-end or at the 5’-end (see Table 1 and Table 2). The osmylated pGEX3’ (R1, 4 dT(OsBp)) exhibits 18-fold or 16-fold slower translocation at 120 or 140 mV, respectively, compared to the control dA_20_ (durations increased from 0.05 to 0.89 ms at 120 mV (Figure 4) and from 0.03 to 0.49 ms at 140 mV). A 0.89 ms translocation for a 23 nt oligo yields an average speed of 40 μs per base, a measurable quantity by current state-of-the art instruments, indicating that the use of a processing enzyme to show down DNA translocation could be avoided. Complete osmylation of both pyrimidines in pGEX3’ R2 (with 4 dT(OsBp) + 5 dC(OsBp)) yields further current obstruction, 11% more blocking, compared to dA_20_ and 3% more blocking compared to pGEX3’ R1. Most likely the low current levels observed with pGEX3’ R2 arise from the sequence region where seven out of eleven nucleotides are osmylated pyrimidines. These data suggest that translocation is sensitive to extent of osmylation, an observation made also with SiN nanopores [50]. The dwell times observed with pGEX3’ R2 are dramatically slow and decrease with increasing voltage. The process at 120 mV is 84-times slower compared to dA_20_ and yields 180 μs per base speed for an oligo with a 39% pyrimidine content.

**Figure 6:**
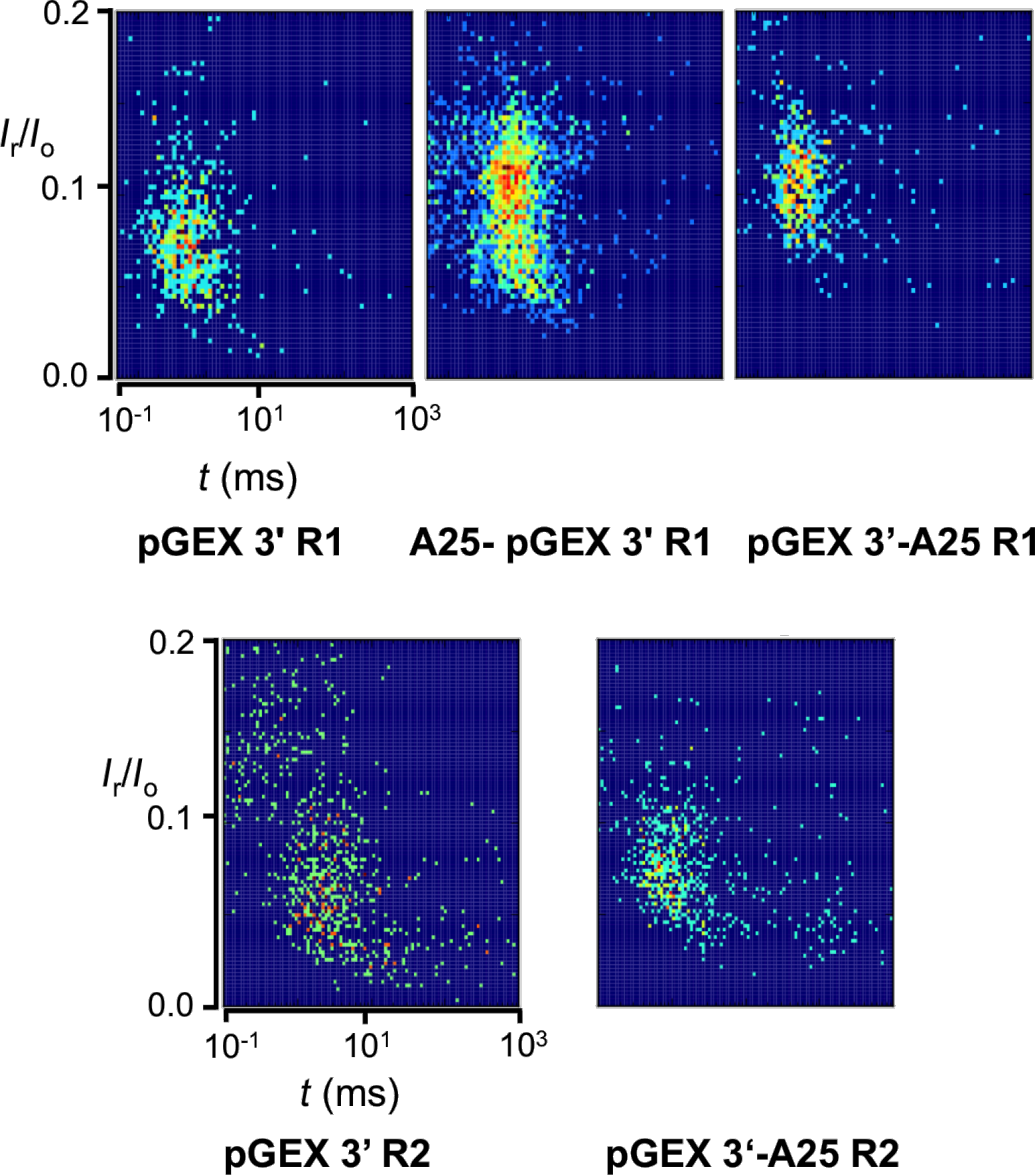
Heat plots, normalized residual current, *I*_r_/*I*_0_, vs translocation time, *t* (ms), for the deoxyoligos with multiple osmylated pyrimidines. pGEX3’ is a 23 nt long PCR primer; R1 stands for 4 dT(OsBp) and R2 stands for {4 dT(OsBp)+5 dC(OsBp)}. Event plots with R1 exhibit a single population. Event plots with R2 exhibit, at least, two populations consistent with other mechanisms operating besides single molecule translocation. pGEX3’-A_25_ R2 exhibits a third population (not shown) at times longer than 1 s. In all cases the A_25_-tail facilitates translocation and more so when it is at the 3’-end compared to the 5’-end.

Notably, the event frequency decreases sharply with osmylation and part of it can be attributed to the slower translocation. Number of events per second are 19, 8, 4, and 0.2 for dA_20_ with no OsBp, dA_10_dT(OsBp)A_9_ with 1 OsBp, pGEX3’ R1 with 4 OsBp and pGEX3’ R2 with 9 OsBp moieties, respectively. In particular the decrease in events frequency between four osmylated dTs and nine osmylated pyrimidines hints to an issue that could be rationalized by complexation of two oligos as follows: Considering that OsBp is a highly hydrophobic moiety, two oligos could associate via hydrophobic interactions along the OsBp moieties (see Figure 5), coexist in the wide vestibule of the α-HL pore, and need to dissociate first before, at least one, translocates. Such phenomenon would sharply reduce the probability to traverse, and even create artifacts like the second population of events observed with the two R2 oligos (see below in Figure 6). It is plausible that this hydrophobic association is favored in the 10 μM oligo concentration and in the presence of 1 M KCl used for the present experiments. Lower oligo and/or lower salt concentrations may suppress association, yield higher frequency of events, a single and more narrowly defined population, and a better understanding of R2 translocation features.

Another way to rationalize the extreme slow-down was to assume the presence of an unidentified impurity in the OsBp preparation that, at the higher concentrations used for Protocol B, yields substantial amounts of cross-linking between strands to form a duplex covalently bound, in a parallel manner, like an “H”. We tested this hypothesis by monitoring the OsBp labeling reaction with a 32 nt long deoxyoligo, pGEX3’-dA_9_ (tail at the 3’-end), by high performance liquid chromatography (HPLC) with ion exchange chromatography (see Experimental section). The oligo was chosen so that cross-linking of two strands to form a 64 nt long conjugate could be easily detected by this HPLC method. The presence of the tail is not expected to have a major effect on the reactivity of the alleged OsBp impurity. Even though we monitored the reaction 3-times longer than protocol B required, there was no detectable formation of longer oligos, with an upper limit of 0.05%, consistent with absence of such impurity.

In an attempt to facilitate translocation and enhance event frequency, we investigated two 48-mers, based on the pGEX3’ sequence, one bearing an dA_25_ tail at the 5’-end and the other bearing the dA_25_ tail at the 3’-end (Table 1 and Table 2). Comparison of the 48-mers at the R1 level with the pGEX3’ R1 indicates that the two longer oligos exhibit comparable current obstruction levels, but obstruct current less compared to the 23-mer. In addition, the dwell times of both 48-mers at 120 mV are shorter compared to the parent oligo (see heat plots in Figure 3). Still the dwell times at 140 and 160 mV are comparable between the 23-mer and the 48-mer with the tail at the 5’-end suggesting that at the higher voltages the tail has no effect on the electrophoretic behavior that is practically determined by the modified sequence. Even though current obstruction is comparable for the two R1 level 48-mers, there is a marked difference in dwell times across all three voltages showing that the 48-mer with the tail at the 3’-end moves almost twice the speed of the 48-mer with the tail at the 5’-end. This behavior is consistent with preference for 3’-entry established experimentally and computationally for native ssDNA [51]. In contrast to the other oligos that exhibit one population in event plots, the R2 level oligos, pGEX3’ R2 and pGEX3’-dA_25_ R2, exhibit two and three, respectively (Figure 6; the third population in pGEX3’-dA_25_ R2 lies outside the graph in the time range). The observation of more than one populations hints to issues with the hydrophobicity of the OsBp moiety discussed above and perhaps with non-optimal experimental conditions. As expected the residual current for these two R2 oligos are lower compared to the corresponding currents observed with the R1 oligos and in the order pGEX3’ R2 < pGEX3’-dA_25_ R2, pointing out that the dA_25_ tail assists in translocation.

**Figure 7:**
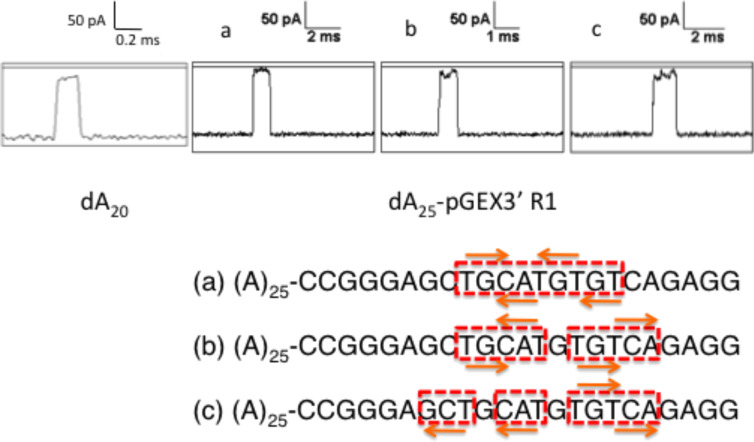
Sample *I*–*t* traces for the control dA_20_ and for dA_25_-pGEX3’ R1 (with 4T(OsBp)) to show (a) continuous blockage, (b) blockage interrupted once, or (c) blockage interrupted twice. These inter-events steps may be attributed to selected configurations (a to c) as above. Theoretically three interruptions of blockages are expected for 4 modifications. Arrows indicate OsBp moiety and have direction; red blocks indicate partial coverage of adjacent bases due to the presence of OsBp. The planar structure of OsBp prohibits full coverage of adjacent bases (not shown in the 2D configuration above). Note that there is more than one plausible configuration to rationalize type b and type c events.

Inspection of the *I*–*t* traces for the pGEX3’-type osmylated oligos revealed many different patterns. Events for dA_25_-pGEX3’ R1 (4 OsBp) exhibited 0, 1, or 2 blockage interruptions or “steps”, see Figure 7. This phenomenon can be rationalized with the help of Figure 5 and Figure 7. Due to its size (estimated at about 0.8 nm) the OsBp moiety extends from the base, that it is attached to, all the way to the third base in the sequence and provides partial “coverage” to two internucleotide spaces (see red blocks in Figure 7). Depending on the direction of the osmylation (orange arrows, top or bottom), the presence of osmylated Ts in a sequence can easily lead to an apparent uninterrupted blockage, as shown schematically in sequence a of Figure 7, blockage interrupted once (sequence b), or blockage interrupted twice (sequence c). Even though the planar structure of OsBp may extend along the strand, it cannot encircle the adjacent base. Hence we speculate that if the overall current blockage was not as severe as it is under our conditions, the intact bases might have made their presence detectable by influencing the current. Optimization of the conditions to produce more current, or change to a wider/shorter pore, such as a solid-state nanopore, may yield better discrimination between an osmylated base and the adjacent intact one. Even using the present conditions one should be able to show experimentally X number of steps in *I*–*t* events for an oligo composed of X + 1 pyrimidines separated by at least five purines.

## Conclusion

In summary, osmylated oligos traverse the α-HL nanopore and block measurably more current compared to the unmodified control. Translocation speeds with osmylated oligos are slow, decrease with degree of osmylation, and reach detectable levels by current state-of-the-art instruments. The observation that the nanopore discriminates by both residual current and dwell time, among a single dT(OsBp), a single dC(OsBp), and a purine, provide proof-of-principle for nanopore-based sequencing using osmylated DNA as a surrogate. Further optimization of the conditions is necessary to suppress hydrophobic complexation between strands and improve discrimination. Obtaining sequence information directly from dsDNA is envisioned as follows: In the presence of a denaturant, dsDNA may be subject to osmylation by protocol B and yield two osmylated ssDNA strands. The osmylated target strand could provide sequence information for T, C, and purine, and the osmylated complementary strand sequence information for A, G, and pyrimidine (bases in the target). Mathematically speaking the system is fully described. Moreover the number of consecutive pyrimidines in the target strand at any location in the sequence should be identical to the number of consecutive purines in the corresponding location of the complementary strand. The last feature gives a handle to partially compensate for top or bottom addition of OsBp to base. The results reported here make the strategy outlined in Figure 1 (ii) testable.

## Experimental

**Part A, Materials, oligos, preparation of osmylated oligos, capillary electophoresis (CE) analysis, and high performance liquid chromatography (HPLC) analysis:** HPCE grade solution of 50 mM sodium tetraborate pH 9.3 was purchased from Agilent Technologies. A 4% aqueous osmium tetroxide solution (0.1575 M in ampules at 2 mL each) was purchased from Electron Microscopy Sciences. 2,2’-Bipyridine 99+% (bipyridine) was purchased from Acros Organics. Oligos were purchased from Integrated DNA Technologies (IDT), diluted with DNase/RNase-free water (from MP Biomedical) to 2 μg/mL or 100 μM and stored at −20 °C. The purity of these oligos was tested using CE in 50 mM sodium tetraborate at pH 9.3 with 20 kV. Oligos used in this study, their sequences and purity are listed in Table 1. The oligo used for the experiment in Figure 2, was a different batch of much better purity compared to the one listed in Table 1.

Analyses were carried out using an Agilent 1600 CE instrument equipped with diode array detector (DAD) and Chemstation software, Rev.B.04.02SP1, for data acquisition and processing. Untreated fused silica capillaries (50 mm × 40 cm) with extended light path were purchased from Agilent Technologies. Monitoring of reactions was conducted using the same CE method as for purity check (see above). For identification purposes we abbreviate R1 (312/272) the ratio of the absorbance at the two wavelengths for the product of the reaction following Protocol A, and R2 (312/272) the corresponding ratio following protocol B (see below). The values R1 and R2 serve as quality control of the product because they can be calculated from the relationships R1 = 2.21 × dT/*N*_total_ and R2 = 2.01 × (dT+dC)/*N*t_otal_, where *N*_total_ is the total number of nucleotides, dT/*N*_total_ is the fraction of dT, and (dT+dC)/*N*_total_ is the fraction of pyrimidines in the sequence [46]. Unpublished kinetic determinations under identical conditions reveal intrinsic selectivity for osmylation of dT/dC = 28 (in agreement with previous data [42]), dT/5-MedC = 6.9, 5-MedC/dC = 4.1, dU/dC = 3.75, and 5-MedC/dU = 1.1.

Osmylated T is stable at room temperature, but osmylated C hydrolyzes/deaminates slowly to form dU [52]. Under our conditions, water at room temperature, the reaction is about 1 to 2% per hour (unpublished results). Protocol B, as described in [46], to effectively osmylate dC takes 11 h, so conversion of C to U would become important, and perhaps mislead the nanopore measurements. We recognized this problem early, and developed a new procedure for OsBp preparation and new protocols [53] that we applied to the preparation of the osmylated oligos in this study. The new protocols are summarized below. The new OsBp preparation is still 15.75 mM in OsO_4_, just as in the old protocols [42,46], but prepared in saturated 2,2’-bipyridine using a 4 to 8-fold molar excess of the later. After vigorous mixing of the two components, the supernatant is removed and used as the new stock solution (OsBp 15.75 mM in saturated 2,2’-bipyridine (sat. bipy)). Saturated 2,2’-bipyridine in water is approximately 30 mM as indicated in the literature. Experiments and kinetic determinations with the new OsBp stock solution (unpublished results) revealed that the reactivity is 4-fold higher compared to the OsBp 1:1 preparation, as described in [42,46]. Hence, we recommend the new protocol A as 60 min incubation in 1.575 mM OsBp (sat. bipy), and the new protocol B as 110 min incubation in 12.6 mM OsBp (sat. bipy). Please note that the stock solution is saturated in bipyridine, because of the way it was prepared. However the resulting reaction mixtures, because they are accordingly diluted (either to 1.575 mM or to 12.6 mM), are no longer saturated in bipyridine. Based on the new reactivities (unpublished results) new protocol A results in approximately 95% T-osmylation and 10% C-osmylation; whereas new protocol B results in over 99.99% T-osmylation and 99.99% C-osmylation. Care was taken that C-osmylated oligos were kept refrigerated/frozen.

HPLC was conducted with an Agilent 1100/1200 HPLC equipped with a binary pump and individual thermostats for autosampler and column compartment. Chemstation software Rev.B.04.01 SP1 were used for data acquisition and processing. The column was a 2 × 250 mm BioLC DNAPac^®^ PA200 from Dionex used in conjunction with 25 mM Tris·HCl pH 7 buffer and a NaCl gradient; the column was maintained at 30 °C. This type of ion-exchange chromatography resolves oligos based on length and composition.

**Part B, Chemicals, materials and instrumentation for nanopore measurements:** Nanopore experiments were conducted with 10 μM DNA in 1.0 M KCl, 10 mM potassium phosphate buffer at pH 7.4 and at 22 ± 1 °C, as described in detail in [14], and summarized here. WT α-hemolysin was purchased from List Biological Laboratories in the monomer form of lyophilized power and dissolved in water at 1 mg/mL. 1,2-Diphytanoyl-*sn*-glycero-3-phosphocholine (DPhPC) was dissolved in decane at 10 mg/mL and used to form the bilayer. The bilayer was supported by a glass nanopore membrane (GNM), which was modified with a 2% (v/v) (3-cyanopropyl)dimethylchlorosilane in acetonitrile to create a moderately hydrophobic surface. Current–time (*I*–*t*) recordings were performed at 22 ± 1 °C using a custom-built high impedance and low-noise system (Electronic BioSciences Inc., San Diego, CA) for the Chemistry Department, University of Utah. The KCl solution was used as the electrolyte to fill the solution reservoir and the GNM capillary. A voltage was applied across the GNM between two Ag/AgCl electrodes placed inside and outside of the capillary. A lipid bilayer was deposited across the GNM orifice as indicated by a resistance increase from ca. 10 MΩ (associated with the open GNM) to ca. 100 GΩ. A pressure of 20 to 40 mmHg was applied to the inside of the GNM capillary using a syringe, allowing the lipid bilayer to be functional for the protein channel reconstitution. Next, 0.2 µL of α-HL monomer solution at 1 mg/mL was added to the *cis* side of GNM (a volume of 350 µL). A voltage of 120 mV (*trans* vs *cis*) was applied. The *I*–*t* traces were filtered at 100 kHz and sampled at 500 kHz. Events were extracted using QuB (version 1.5.0.31), and histograms were analyzed by Origin 9.1. Heat plots were plotted using data analysis programs provided by Electronic BioSciences Inc., San Diego, CA.
